# Primary vitreoretinal lymphoma: diagnosis, treatment, and prognosis—a review of current knowledge and future directions

**DOI:** 10.1097/BS9.0000000000000233

**Published:** 2025-05-01

**Authors:** Si-Yu Wang, Suo-Wang Zhou, Jing Gao, Liang Wang

**Affiliations:** a Capital Medical University, Beijing, China; b Aier Eye Hospital, Jinan University, Guangzhou 510071, China; c Department of Hematology, Beijing Tongren Hospital, Capital Medical University, Beijing, China

**Keywords:** Autologous stem cell transplantation, Bruton’s tyrosine kinase inhibitors, Methotrexate, Primary central nervous system lymphoma, Primary vitreoretinal lymphoma

## Abstract

Primary vitreoretinal lymphoma (PVRL), a rare subtype of primary central nervous system lymphoma (PCNSL), can lead to permanent vision loss and central nervous system (CNS) involvement, resulting in a poor prognosis. PVRL often masquerades as uveitis, and its partial response to topical corticosteroids further complicates the diagnosis. The gold standard for diagnosis is cytological analysis; however, owing to its low sensitivity, cytokine profiling and genetic testing may serve as supplementary diagnostic tools. There is no universally accepted consensus regarding PVRL treatment protocols. Combined systemic high-dose intravenous methotrexate (MTX) and intravitreal therapy may help manage bilateral ocular lesions, although this combination’s ability to delay CNS relapse remains controversial. For relapsed or refractory (R/R) PVRL patients aged <60 years, intensive consolidation chemotherapy followed by autologous stem cell transplantation may be considered. Novel targeted therapies such as ibrutinib and lenalidomide have demonstrated efficacy in R/R cases. Large-scale multicenter prospective studies are urgently needed to determine optimal treatment strategies.

## 1. INTRODUCTION

Primary vitreoretinal lymphoma (PVRL) is a rare form of non-Hodgkin lymphoma that is limited to intraocular tissues, approximately 95% of which are diffuse large B-cell lymphomas (DLBCL).^1^ PVRL is a subtype of primary central nervous system lymphoma (PCNSL), which is distinguished from systemic lymphoma.^2^ PVRL can be fatal in cases of central involvement. Because PVRL often masquerades as uveitis, it can be difficult to diagnose at an early stage, causing central nervous system (CNS) involvement.^1^ Considering the correlation between PVRL and PCNSL, as well as the presence of the blood-retinal barrier, central and local therapies should both be involved in the treatment. However, the low incidence of PVRL makes it difficult to conduct diagnostic and therapeutic studies, exacerbating the poor prognosis of the disease. This review concludes with both traditional and emerging methods for the diagnosis, treatment, and prognosis of PVRL, as well as the advantages and limitations of each, to identify the future challenges for further research into this disease.

## 2. EPIDEMIOLOGY

PVRL is one of the rarest primary ocular tumors and its incidence is gradually increasing. The overall age-adjusted incidence of PVRL in the US is 0.23/1,000,000, with an increasing trend over three decades.^3^ This growing trend may be associated with an increasing number of immunodeficient and immunosuppressed patients, increased life expectancy, and advances in diagnostic methods.

Elderly individuals are at a high risk of developing PRVL. The median age at diagnosis is over 63 years^4^ and more than 20% of patients are over 80 years old.^5^ A retrospective analysis over a period of 20 years showed that the incidence of PVRL ≥60 years individuals is nearly 18 times higher than in <60 years individuals.^3^ Human immunodeficiency virus (HIV) and Epstein-Barr virus (EBV) infection are important risk factors, and their incidence is inversely correlated with CD4 count.^6^ However, the impact of acquired immunodeficiency syndrome (AIDS)-related immunodeficiencies may be less important in this age of highly effective antiretroviral therapies. There are no other known risk factors for PVRL.

## 3. CLINICAL FEATURES

The clinical manifestations of PVRL are non-specific, with the main symptoms including floaters and painless vision loss, similar to the typical presentation of refractory uveitis.^7^ However, patients with PVRL are older, have lower visual acuity at presentation, and are less likely to be female than those with uveitis, which might help distinguish PVRL from uveitis.^8^ PVRL predominantly presents with bilateral ocular involvement. In a retrospective study, 50% to 80% of cases showed bilateral involvement, but the degree of lesions in both eyes could be asymmetrical.^7^

PVRL may be accompanied by neurological symptoms preceding the onset of brain lesions. The most common related neurological symptoms include behavioral and/or cognitive changes in 25% to 30% of patients, hemiplegia in 10% to 15%, headaches in 10% to 15%, aphasia in 10% to 15%, seizures in 5%, and ataxia in 4%, and these symptoms usually persist for weeks to months.^8,9^

The pathology of VRL is characterized by the invasion of intraocular tissues such as the vitreous, retina, anterior chamber, and optic nerve by tumor cells, often with anterior segment inflammation.

**Figure 1.**
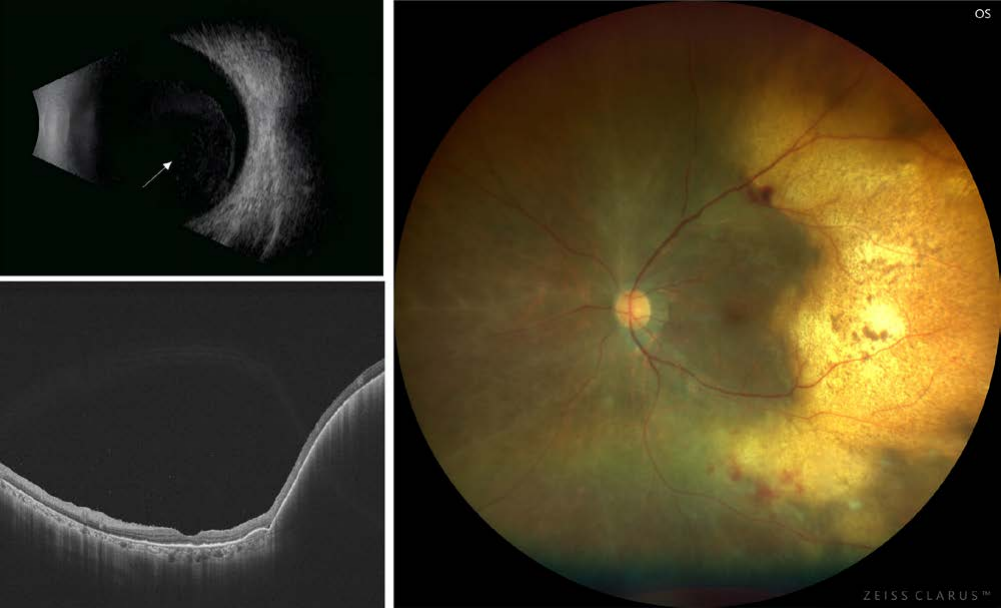
Multimodal images of a 75-y-old female diagnosed as vitreoretinal lymphoma. Left top: Ocular B-scan ultrasonography reveals diffuse posterior vitreous haze. Left bottom: OCT shows confluent RPE detachment temporal to the macula with homogeneous hyperreflective mass beneath it. Right: Ocular fundus photograph displays an annular yellowish-white elevated lesion temporal to the macula accompanied by small pigment clumps on the surface. OCT = optical coherence tomography, RPE = retinal pigment epithelium.

Vitreous opacity can manifest as a “Northern Lights” pattern, with numerous cells lining the vitreous fibrils.^10,11^ Vitreous opacity can also be described as veil patterns with delicate textures that extends radially.^12^ These lesions can be observed using ultrawide-field fundus imaging. In addition, ophthalmologic ultrasonography has shown that the erythrocyte material was associated with vitreous opacity^10^ (**Fig. 1**).

Subretinal yellowish lesions are an important clinical feature of PVRL as revealed by ultrawide-field fundus imaging. This lesion appears on optical coherence tomography (OCT) as highly elevated retinal pigment epithelium (RPE) layer and “vertical hyperreflective column” (VHRL), which is considered to represent micro-invasion of the tumor^13,14^ (**Fig. 1**). Fundus autofluorescence (FAF) shows hyperautofluorescence, which corresponds to the areas of subretinal lesions and nodular hyperreflex points on OCT image.^15^

Retinal infiltration is an important clinical feature in PVRL. Color fundus photographs showed white retinal infiltrates with vitreous opacity, optic nerve edema, perivascular sheaths, RPE lesions, and serous retinal detachment^14,16^ (**Fig. 1**). Because lymphomatous RPE infiltration alters RPE metabolism, FAF images appear to be hyper-fluorescent.^17^ In contrast, areas of lymphoma infiltration above the RPE appear to have low autofluorescence in FAF, possibly because of tumor blocking of normal RPE fluorescence.^15^ Low autofluorescence is also observed in cases of RPE atrophy after the spontaneous resolution of retinopathy.^18^ OCT revealed hyperreflective subretinal lesions that regressed or progressed to subretinal fibrosis after treatment.^19^

## 4. DIAGNOSIS

**Figure 2.**
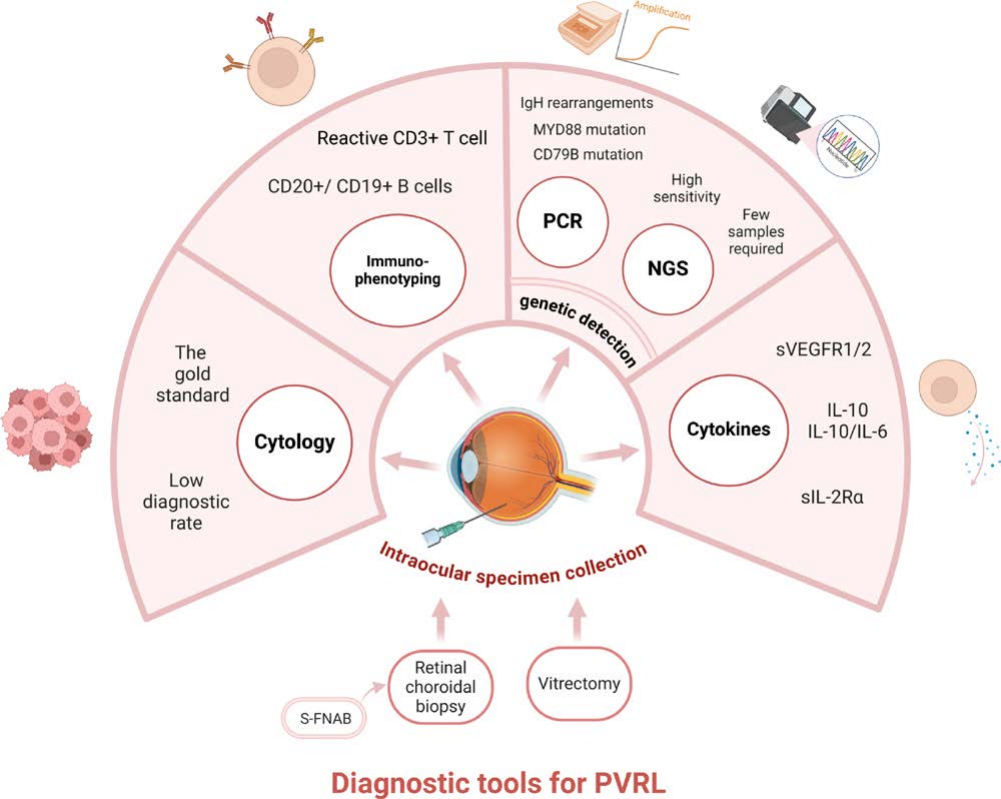
The current tools for diagnosing primary vitreoretinal lymphoma. MYD88 = myeloid differentiation primary response 88, NGS = next-generation sequencing, PCR = polymerase chain reaction, PVRL = primary vitreoretinal lymphoma, S-FNAB = silicone fine-needle aspiration biopsy, sIL-2Rα = soluble IL-2 receptor α, sVEGFR = soluble vascular endothelial growth factor receptor. This image was created using BioRender.

In patients with a clinical suspicion of PVRL, noninvasive ocular and CNS examinations should be performed. Magnetic resonance imaging (MRI) may be the preferred test for the CNS, along with a lumbar puncture, to obtain a cerebrospinal fluid specimen (CSF) from which lymphoma cells are found to confirm the diagnosis of PCNSL.^20,21^ It is of little significance to perform an intraocular biopsy after the diagnosis of PCNSL and a positive ocular imaging test.^22^ An intracranial biopsy may be indicated in patients with negative CSF cells and radiographic suspicion of intracranial lesions.^23^ If no CNS involvement is observed, an intraocular sample is collected for testing (**Fig. 2**).

### 4.1. Intraocular specimen collection

Depending on the amount of tumor cell infiltration, the most common site for ocular biopsy is the vitreous, and vitrectomy is preferred because it is less invasive than retinal or uveal biopsy.^14^ Undiluted samples are obtained by dry vitrectomy for cytological and cytokine analysis.^12,14^ Subsequently, the residual vitreous is removed by vitrectomy to collect diluted specimens and improve visual acuity.^24^ The diluted samples are used for immunoglobulin gene rearrangement, gene mutation analysis, bacterial or fungal culture and staining, and polymerase chain reaction (PCR) detection of various microorganisms.^12^ Given the lymphocyte-suppressive and lytic effects of glucocorticoids, patients should stop taking glucocorticoids in the weeks before vitreous biopsy.^1,25^

If no or few tumor cells are found in the vitreous, retinopathy suggests that PVRL and retinal choroidal biopsy are recommended. A recent study showed that retinal choroidal specimens can be obtained using 41-gauge silicone fine-needle aspiration biopsy (S-FNAB).^26^ The needle is very thin (0.0711 mm) and flexible enough to penetrate the retina and subretinal space to aspirate lymphoma cells at the lesion.^26^ Compared with other retinal choroidal biopsy methods, S-FNAB does not significantly increase retinal thickness and allows macular area sampling.^26,27^

### 4.2. Cytological examination

Multiple studies have shown that cytology is the gold standard for diagnosing VRL.^28^ There are 2 types of cytological examination methods: conventional smear cytology and cell block preparation, with the former having a higher positive rate.^29,30^ Given the high specificity based on morphological analysis and immunocytology, the blockade method can be used for the differential diagnosis of VRL and uveitis.^30^ Lymphoma cells are atypical large lymphocytes with irregular nuclei, multiple distinct nucleoli, and sparse cytoplasm, usually accompanied by small reactive T lymphocyte infiltration.^31^ Although vitreous cytology is the gold standard for diagnosis, a negative result does not definitively rule out PVRL because of its low diagnostic rate.^11,25,32^ The low diagnostic rate may be due to the following reasons: inexperienced ocular pathologists, limited sample size, low number of cells in the sample, fragile intravitreal lymphoma cells, and contamination with other cellular structures.^33^

### 4.3. Immunohistochemistry or flow cytometry

Atypical monocytes on cytological examination may also be present in a reactive state; therefore, further examination of B/T-cell surface markers by immunohistochemistry is required.^11^ Because most PVRL are derived from B lymphocytes, immunohistochemistry can detect CD20+ and CD19+ B cells as well as reactive CD3+ T cells.^34^

Flow cytometry is another reliable cell phenotyping test. It detects a large number of cell surface markers that can differentiate PVRL from uveitis.^35^ Moreover, the imbalance in the ratio of Igκ to λ (<0.1 or >10) is regarded as the marker for clonality of B-cell tumor cells.^35^

### 4.4. Genetic mutations detected by PCR

#### 4.4.1. IgH rearrangements

Positive immunoglobulin heavy chain (IgH) gene rearrangements can be detected in patients with PVRL, and some can detect positive T-cell antigen receptor (TCR) gene rearrangements, which are helpful for identifying the monoclonal and neoplastic properties of lymphocytes.^36^ The largest study on this test was published by the U.S. National Institutes of Health (NIH) in 2011, in which they tested samples from 200 patients with uveitis masquerade syndrome (114 with confirmed PVRL and 86 with confirmed uveitis) and showed that either IgH (109) or TCR (5) gene rearrangements (sensitivity, 100%; specificity, 99%) were present in all patients.^37^ Among the 86 patients with uveitis, 1 patient was positive for IgH gene rearrangement.

Chromosomal rearrangements can also relocate to specific gene positions. The translocation of t(14:18) moves the Bcl-2 gene, which encodes an anti-apoptotic molecule, to chromosome 14, resulting in the overexpression of Bcl-2 in B cells, thus prolonging their survival.^38^ In another study conducted by the NIH, t(14:18) rearrangements were identified cytologically in 41 of 72 patients with confirmed vitreoretinal lymphoma cytologically.^39^

#### 4.4.2. Mutations in MYD88 and CD79B

Myeloid differentiation primary response gene 88 (MYD88), which encodes a universal adaptor protein used by Toll-like receptors in the innate immune system, is frequently mutated in DLBCL, especially at immune-privileged sites, such as the CNS.^40,41^ Approximately 69% to 88% of patients have MYD88 mutation.^42,43^ If the MYD88 allele mutation is present in the CD20+ cells in the vitreous humor, a diagnosis of PVRL can be confirmed.^1^ MYD88 mutation analysis is often used as an additional tool for the early diagnosis of PVRL. In the early stages of the disease, insufficient cells in vitreous samples make cytological evaluation difficult. Moreover, many patients are treated with steroids owing to the misdiagnosis of vitritis, which can lead to the lysis of tumor cells.^44^ MYD88 PCR requires only 4.93 ng/mL (or close to 20,000 cells) for results and is not dependent on the presence of cells.^42^ Compared with IgH PCR, the detection of MYD88 mutations requires fewer cells, has a lower false-negative rate, and the results are more objective.^33^

The mutation of CD79B, encoding the Igβ protein of the B-cell receptor (BCR), is another supporting diagnostic indicator in patients with PVRL.^45^ Yonese et al^46^ found that the positive rate of CD79B mutations in PVRL was 35%, which is much lower than MYD88 mutation. Thus, CD79B mutations may be associated with CNS progression in PVRL.^46^ In the above study, all patients carrying the CD79B mutation eventually developed CNS diseases despite receiving systemic methotrexate (MTX).

### 4.5. The emerging molecular diagnostic tool: next-generation sequencing (NGS)

NGS is an emerging molecular diagnostic tool that can guide the development of precise molecular-targeted therapies for PVRL. A study that performed NGS analysis of the vitreous humor from 23 patients with VRL showed mutations in MYD88 (91%), CDKN2A (36%), PIM1 (32%), IGLL5 (27%), and ETV6 (23%), with the highest frequency of the MYD88 mutation.^47^ Mutations in MYD88, CDKN2A, PIM1, IGLL5, and ETV6 can be considered important mutations in the VRL family.^48^ In a comparative NGS analysis of brain and vitreous samples from patients with PCNSL and VRL, both samples were found to contain a mutation and clone in MYD88 L265P and a complete deletion of CDKN2A.^49^ These similar genetic mutations suggest a common origin of both tumors.

Vitreous NGS detection had the highest sensitivity (0.85) among previously proposed diagnostic criteria, while the interleukin (IL)-10/IL-6 ratio showed the second highest sensitivity (0.83).^47^ In a study by Cani et al,^50^ NGS successfully detected mutations in cases in which cytology repeatedly showed false negatives, suggesting that NGS may improve prognosis through early diagnosis and treatment. NGS can provide accurate results despite the high viscosity, poor cell preservation, and low cellularity of the sample, which make PCR results false negatives or false positives.^37,50^ In addition, NGS analysis can be performed with only a small amount (as low as 500 μL) of vitreous samples, leaving enough samples for cytology and other diagnostic purposes.^50^

### 4.6. Cytokines

#### 4.6.1. IL-10 and IL-10/IL-6 ratio

IL-10 is expressed by malignant B lymphocytes, whereas IL-6 is mainly expressed by inflammatory cells.^51^ Patients with PVRL have higher levels of IL-10 in aqueous humor or vitreous samples, which are much higher than those in patients with uveitis. An IL-10:IL-6 ratio greater than 1.0 is highly suggestive of lymphoma, as opposed to a ratio of less than 1.0 indicating uveitis.^7,51^ Cassoux et al^52^ defined IL-10-specific thresholds in the intraocular fluid as 50 pg/mL for the aqueous humor (89% sensitivity and 93% specificity) and 400 pg/mL for the vitreous humor (80% sensitivity and 99% specificity). Therefore, the concentration of IL-10 and the IL-10: IL-6 ratio can be used to assist in the diagnosis of PVRL and distinguish it from uveitis.

In a study by Lee et al,^12^ a significant increase in IL-6 levels was detected in three PVRL patients with extensive and severe sub-RPE infiltration, which may be related to disruption of the blood-retinal barrier or recruitment and secretion of reactive cells. For a variety of reasons, the precise thresholds for IL-10 concentration or the IL-10:IL-6 ratio may vary between laboratories.^16^ Considering these limitations, cytokine analysis alone cannot be used to diagnose PVRL.

#### 4.6.2. Soluble vascular endothelial growth factor receptor (sVEGFR)1 and VEGFR2

Vascular endothelial growth factor (VEGF)-A can promote mitosis in DLBCL cells through the VEGFR1 and VEGFR2 pathways.^53^ Takeda et al^54^ showed that both sVEGFR1 and sVEGFR2 levels were significantly higher in patients with systemic metastatic retinal lymphoma (SMRL) than in those with PVRL/PCNSL. Therefore, the measurement of sVEGFR1 and/or sVEGFR2 levels in vitreous fluids is helpful in the differential diagnosis of PVRL/PCNSL and SMRL.

#### 4.6.3. Soluble IL-2 receptor α (sIL-2Rα)

sIL-2Rα prevents the growth of T cells through multiple pathways, including CD8 T cells with anti-tumor activity.^55,56^ The level of sIL-2Rα in patients with VRL was significantly higher than that in patients with uveitis and control groups (*p* < 0.05).^54^ In addition, VRL patients with subretinal lesions and vitreous opacity had higher sIL-2Rα levels than those with predominantly vitreous opacity (*p* = 0.0027).^54^ These results suggest that sIL-2Rα may be a diagnostic marker for VRL and uveitis and an adjunct to the prediction of retinal and/or subretinal malignant lymphoma cell infiltration for early detection and treatment to maintain vision.

## 5. TREATMENT

**Figure 3.**
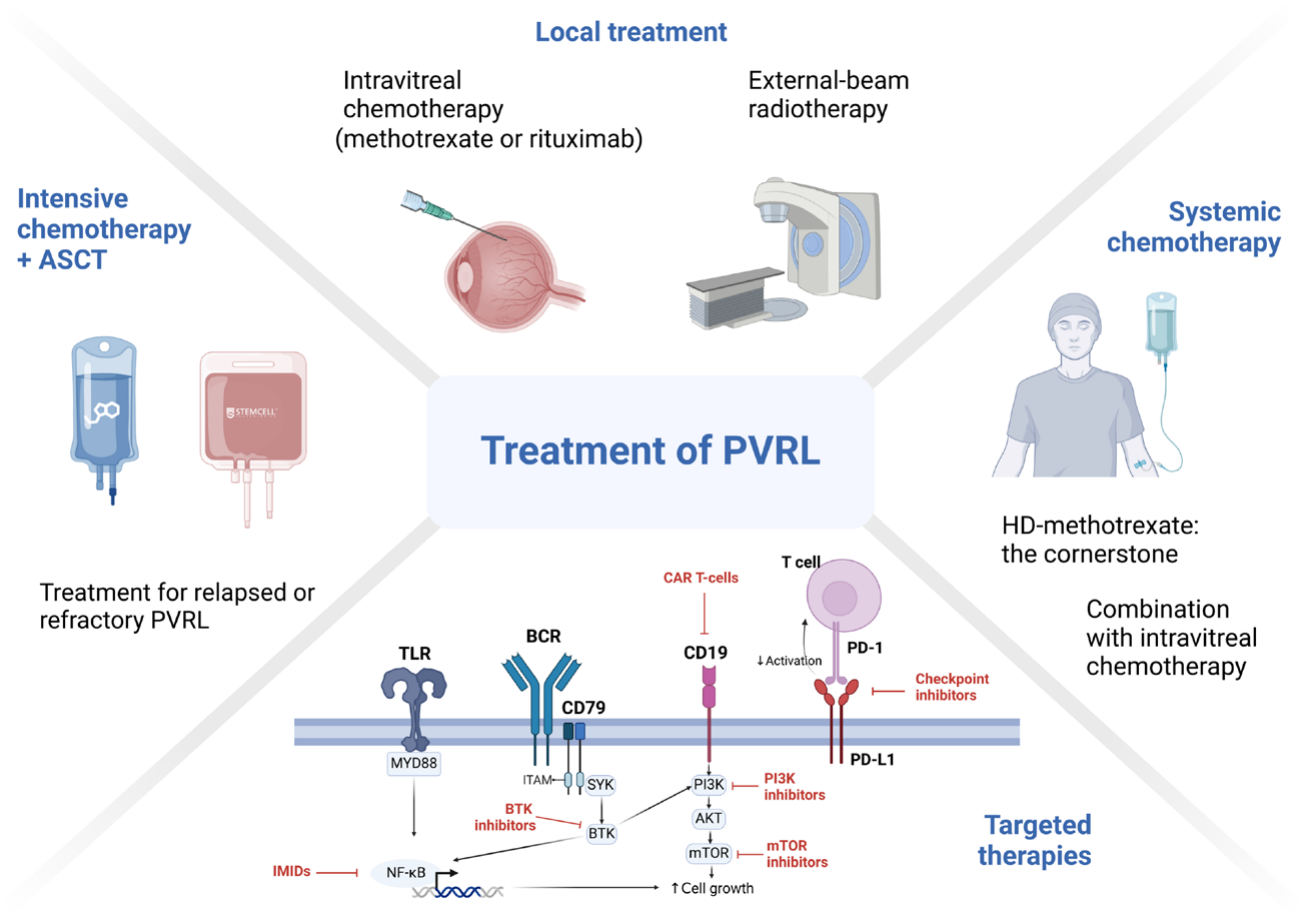
Overview of treatments for primary vitreoretinal lymphoma. Therapies that target certain pathways in primary central nervous system lymphoma highlighted in red are also promising in PVRL. AKT = protein kinase B, ASCT = autologous stem cell transplantation, BCR = B-cell receptor, BTK = Bruton’s tyrosine kinase, CAR = chimeric antigen receptor, HD = high-dose, ITAM = immunoreceptor tyrosine-based activation motif, mTOR = mechanistic target of rapamycin, MYD88 = myeloid differentiation primary response 88, NF-κB = nuclear factor kappa-light-chain-enhancer of activated B cells, PI3K = phosphoinositide 3-kinase, SYK = spleen tyrosine kinase, TLR = toll-like receptor. This image was created using BioRender.

The goal of treatment is to eradicate intraocular disease and prevent CNS lymphomas. To date, no established treatment methods have been identified for PVRL. The use of local treatment alone (such as ocular irradiation or intravitreal chemotherapy with MTX or rituximab) or in combination with systemic chemotherapy remains controversial. For relapsed or refractory (R/R) cases, autologous stem cell transplantation (ASCT) along with targeted therapies (such as lenalidomide or ibrutinib) is recommended (**Fig. 3**).

### 5.1. Local ocular treatment

Local ocular treatments include intravitreal MTX, intravitreal rituximab (IVR), and external beam radiotherapy (EBRT). The intravitreal MTX injection dose is 0.4 mg/0.1 mL.^28^ The injection cycle during the induction period is performed twice weekly for 4 weeks.^28^ Subsequent injections can be performed as predetermined (a total of 25 intravitreal injections over 1 year) or adjusted according to the clinical response and aqueous humor IL-10 levels.^28^ A large-scale retrospective analysis of 81 patients with VRL (134 eyes) highlighted the efficacy and safety of intravitreal MTX therapy for more than 20 years.^57^ The mean number of injections administered was 19. However, complete remission (CR) was achieved after an average of 5 injections. The criterion for CR was the disappearance of lymphoma cells in the vitreous cavity as observed using a slit lamp. Intravitreal injection of MTX can achieve high concentrations of intraocular MTX, which ensures effectiveness; however, it can also cause ocular side effects. Corneal lesions were the most common side effect, occurring in all patients during the first 5 injections. Fortunately, this can be mitigated by various methods, including reducing the volume of MTX and extending the interval between injections. Therefore, intravitreal injection of MTX has been shown to be not only effective but safe for the treatment of VRL.

Recently, IVR has emerged as an intraocular local alternative. Rituximab, an anti-CD20 monoclonal antibody, induces apoptosis in tumor cells by targeting B-cell surface antigens. IVR was administered at 1 mg/0.1 mL.^1^ The injection cycle for IVR varies widely and protocols have not been established for large cohorts of patients with PVRL. Rishi et al^58^ evaluated the treatment outcomes and complications of IVR as a monotherapy for VRL. Complete regression was observed in 12 eyes of all 7 patients with VRL. The main complications were anterior uveitis (50%) and increased intraocular pressure (25%). However, in the study by Larkin et al’s,^59^ only 31 of 48 eyes treated with IVR (64.6%) achieved CR after a median of 3 injections, and seven eyes developed recurrent disease. As most studies on IVR therapy are case reports or small case analyses, the efficacy of this approach cannot be proven. Overall, IVR injection demonstrated comparable efficacy to MTX, with the advantage of lower local toxicity, such as a reduced incidence of keratopathy compared with MTX.^60^

EBRT typically involves a total of 35 to 40 Gy, with a median efficacy of 36 Gy being the most satisfactory.^1,61^ ERBT is particularly effective in bilateral PVRL lesions but cannot prevent CNS recurrence.^61^ Cataracts and radiation retinopathy are common side effects of EBRT.^1,62^ The incidence of retinopathy increases with high dose.^62^ Cataracts may decrease the sensitivity of retinal examination, leading to missed diagnosis of mild retinopathy.^1^

### 5.2. Systemic chemotherapy

High-dose MTX (HD-MTX) is considered the cornerstone of systemic chemotherapy for newly diagnosed PCNSL. Based on the anatomical and functional similarities between the blood-brain barrier and BRB,^63^ systemic chemotherapy with HD-MTX is used as an empirical treatment in patients with PVRL. In a prospective study conducted by Kaburaki et al,^64^ 11 patients with PVRL without CNS involvement received systemic intravenous rituximab, MTX, procarbazine, and vincristine (R-MPV), followed by reduced-dose whole-brain radiotherapy (rdWBRT) and intravenous HD-cytarabine. In these 11 patients, the cumulative incidence of 4-year CNS progression was only 10.0%, and the 4-year PFS was 72.7%. This study indicates that prophylactic rdWBRT combined with systemic chemotherapy can effectively inhibit CNS metastasis and improve prognosis.^64^ A retrospective study conducted by Hashida et al^65^ concluded that prophylactic systemic chemotherapy, particularly HD-MTX, did not reduce the rate of CNS involvement in most patients with PVRL, but significantly prolonged the duration of brain involvement. Thus, systemic prophylactic chemotherapy may improve the prognosis by prolonging the progression-free period.

Although systemic HD-MTX chemotherapy has been shown to achieve cytotoxic levels in the vitreous and aqueous humors, low concentrations of the drug in the vitreous water may lead to the persistence or recurrence of PVRL in some patients. Intraocular chemotherapy did not prevent recurrence. Systemic chemotherapy combined with intravitreal chemotherapy may compensate for these deficiencies. To date, only one prospective study has reported the effectiveness of a combination of intravitreal MTX and systemic chemotherapy in preventing CNS progression.^66^ All patients with PVRL achieved CR after the initiation of intravitreal MTX injection. The 2-year CNS lymphoma-free survival (CLFS) of patients receiving intravitreal MTX in combination with systemic HD-MTX was 58.3% compared with 37.5% of patients receiving intravitreal MTX alone. Although CLFS was longer in patients receiving combination chemotherapy, the comparison is not straightforward because of the differences in their backgrounds (median age, etc). Paradoxically, a retrospective cohort analysis of 70 patients with PVRL who received intraocular and systemic chemotherapy concluded that the mean time to CNS involvement and death did not differ according to the primary method of PVRL treatment. Additionally, multicenter retrospective studies have shown no efficacy of systemic chemotherapy in CNS recurrence.^67,68^ Nevertheless, large prospective multicenter studies are required to confirm the effectiveness of intravitreal and systemic combination chemotherapy.

### 5.3. Treatment for R/R PVRL

Data on intense chemotherapy (IC) and ASCT for the treatment of PVRL are limited, with most trials including either PCNSL alone or both PCNSL and PVRL. In a prospective, multicenter trial conducted by Soussain et al,^69^ 27 patients with R/R PCNSL or VRL were treated with IC + hematopoietic stem-cell rescue (HCR). The IC regimen, which combines thiotepa, busulfan, and cyclophosphamide, has been shown to be effective in patients with systemic lymphoma with a poor prognosis, including those with CNS lymphoma involvement.^70^ The results showed that all but one patient achieved a complete response. Another study conducted by Soussain et al^71^ revealed similar results, with 16 of 20 patients with R/R PCNSL or VRL treated with IC + HCR entering CR, 2 maintaining PR, 1 with stable disease, and 1 with disease progression. Both studies demonstrated that IC + HCR was effective for R/R VRL. However, a LOC network study by Mainguy et al^72^ showed that despite favorable survival outcomes, the rate of recurrence after IC-ASCT, particularly CNS relapse, remained high in patients with PVRL and PCNSL at initial diagnosis. Regular brain examinations should be performed after IC-ASCT. A retrospective study involving 79 patients with R/R PCNSL or VRL found that age was an independent factor of overall survival (OS).^73^ The combination treatment was feasible in patients under 60 years of age, but could also be tolerated in fit patients above 60 years of age by reducing the busulfan dose.

### 5.4. Emerging targeted therapies

#### 5.4.1. Lenalidomide plus rituximab

Lenalidomide is a potent anti-proliferative and immunomodulatory agent that has been shown to be effective in combination with rituximab for treating recurrent CNS lymphoma.^74^ A prospective phase II study conducted by Ghesquieres et al^75^ evaluated lenalidomide in combination with intravenous rituximab (R2) in patients with R/R PCNSL or PVRL. Nine patients with PVRL and 10 patients with secondary VRL were included in this study. The results showed that 35% (n = 9) of patients achieved CR. Thus, the R2 regimen can be considered an alternative therapy for patients with R/R PVRL who are unfit for more intensive salvage treatment. In another prospective study conducted by Zhang et al,^76^ 11 patients with PVRL were treated with the R2 regimen combined with intravitreal MTX, followed by lenalidomide maintenance. At the first evaluation, 10 of the 11 patients achieved CR. However, 8 patients relapsed, including 5 with CNS and 3 with intraocular recurrence. This study suggests that the addition of the R2 regimen to intravitreal MTX injections is a safe and effective option for treating PVRL. CSF IL-10 concentration should be monitored to detect recurrence in the early phase.

#### 5.4.2. Bruton’s tyrosine kinase inhibitors (BTKi)

Mutations in CD79B and MYD88 have been detected in the vitreous samples of most patients with VRL.^77^ These mutations enhance BCR signaling and promote B-cell activation and survival.^77,78^ Bruton’s tyrosine kinase (BTK) is a key enzyme in the BCR pathway, promoting the nuclear translocation of nuclear factor kappa B (NF-κB), thereby leading to B-cell proliferation. Therefore, BTKi can downregulate the BCR pathway and inhibit the proliferation of malignant B cells. BTKi has been used clinically as a targeted drug for malignant B-cell lymphoma; however, its efficacy in patients with PVRL has not been determined. To further evaluate the efficacy of ibrutinib (a first-generation BTK inhibitor approved for marketing) as a single agent for treating patients with VRL, Guan et al^79^ designed a single-center, prospective phase II clinical trial. Among the 10 patients with VRL included in the study, 9 (90%) achieved disease control (DC) after 1 month of treatment, 7 (70%) experienced CR of symptoms, and 2 (20%) experienced partial remission. This indicates that the BTKi treatment of VRL is feasible. Gao et al^80^ compared single-agent BTKi treatment to combination therapy. The objective response rate (ORR) was similar between the BTKi monotherapy and combination therapy groups (96% vs 89%); however, the CR rate was lower in the BTKi monotherapy group than in the combination therapy group (79% vs 92%). The incidence of grade 3/4 toxicities was higher in the combination therapy group (45%). Therefore, the combination of BTKi with various treatment regimens may represent a future trend. However, with the widespread use of these drugs, resistance to BTKi has also emerged. ABC-DLBCL with MYD88^L265P^ and CD79A/B mutations responded to ibrutinib (80% response rate); however, tumors with MYD88^L265P^ mutations without CD79A/B mutations were resistant to ibrutinib,^81^ which indicated that MYD88^L265P^ mutations may be associated with resistance to ibrutinib.

## 6. T CELL OR NK/T-CELL PVRL

Most PVRLs are DLBCL, and those of T cell or NK/T-cell origin are rare. When a patient presenting with uveitis does not respond to steroid therapy, and B-cell PVRL is excluded, the possibility of T cell or NK/T-cell lymphoma should be considered. Cytological examination remains the diagnostic gold standard for T-cell PVRL, with hematoxylin and eosin (HE) staining typically revealing medium-to-large lymphocytes with atypical nuclei.^82^ Immunohistochemical testing shows that lymphocytes expressed CD3 but not CD20.^82^ Genetic testing shows TCR gene rearrangements.^83^ Cytological examination of NK-cell PVRL revealed medium-sized neoplastic cells with distorted nuclear nuclei.^84^ Immunohistochemistry demonstrates CD3 and CD56 positivity as well as EBV-encoded RNA.^84^ EBV can be detected through PCR analysis of aqueous humor samples.^85^ Given that IL-10 is mainly secreted by B lymphocytes, cytokine analysis of either T cell or natural killer (NK)/T-cell type PVRL showed lower concentrations of IL-10.^85,86^

As most studies on T cell or NK/T-cell PVRL are case reports or small retrospective case studies, there is currently no recommended treatment protocol. Treatment is mainly administered according to the therapeutic approach for DLBCL PVRL, such as intravenous or intravitreal injections of MTX.

## 7. PROGNOSIS

Several studies have reported a poor prognosis for PVRL with CNS involvement.^87–89^ Unfortunately, the rate of CNS involvement in PVRL is relatively high. Approximately one-third of patients with PVRL are diagnosed with PCNSL at presentation, and 42% to 92% progress to PCNSL within an average of 8 to 29 months.^90^ The rate of CNS involvement is influenced by different clinical features. In patients with isolated PVRL, VRL recurrence, elevated IL-10 levels, and sub-RPE infiltration are associated with a high CNS involvement rate.^91^ Elevated IL-10 levels in the aqueous humor are related to high tumor burden and disease activity. Sub-RPE infiltration is highly aggressive, with which patients have a shorter survival time. CNS involvement and VRL recurrence are mutually influencing risk factors in terms of disease progression. Genetic factors can effectively predict CNS progression in patients with PVRL. Yoshifuji et al^92^ demonstrated that ETV6 loss and PRDM1 alterations were the primary risk factors associated with CNS progression in PVRL. Patients can be stratified into slow-, intermediate-, and rapid-progression groups (0, 1, and 2 factors, respectively) based on these 2 genetic factors, thereby guiding clinical management. As mentioned earlier, systemic prophylaxis with MTX chemotherapy may delay CNS disease progression and improve prognosis. Given that PVRL is highly malignant and has a high mortality rate, systemic prophylactic chemotherapy is commonly recommended to delay disease progression without causing significant adverse effects. However, some clinical characteristics such as sex, bilateral ocular involvement, and retinal invasion have not been proven to be prognostic factors for PVRL.^93^ By assessing these prognostic factors, clinicians can screen patients with high-risk PVRL for specific treatments to prolong their survival.

## 8. CONCLUSIONS AND FUTURE DIRECTIONS

Ophthalmologic imaging examinations, such as FAF and OCT, should be performed to distinguish PVRL from uveitis. The development of artificial intelligence (AI) may improve the imaging-based diagnosis of PVRL. Compared with AI, clinicians have limited capabilities and inevitable inconsistencies. AI is likely to deliver more objective diagnostic outcomes through deep learning and modeling of large volumes of ophthalmologic imaging data. Although cytological analysis of vitreous samples remains the gold standard for diagnosis, its sensitivity is limited. Additional tests, including IgH gene rearrangement analysis, detection of the MYD88^L265P^ mutation, and cytokine profiling, can be combined to increase the specificity and sensitivity of PVRL diagnosis.

Given the rarity of the disease, there is still no consensus on the optimal treatment strategies for PVRL, as large-scale prospective studies are lacking. The emergence of targeted therapies has brought hope to patients with PVRL. BTK inhibitors and immunomodulatory agents (eg, lenalidomide), as targeted drugs acting on the BCR signaling pathway, have demonstrated favorable efficacy in clinical trials. Additionally, novel targeted therapies, such as phosphoinositide 3-kinase (PI3K)/mechanistic target of rapamycin (mTOR) inhibitors, along with immunotherapies, including CAR T-cell therapy and immune checkpoint inhibitors, are increasingly applied in the treatment of PCNSL.^94^ These approaches demonstrate the potential efficacy of PVRL with CNS involvement, although this hypothesis requires validation in clinical trials. Further exploration of the pathogenesis of PVRL and detailed individual molecular-level analyses in patients with PVRL may provide novel insights for the development of advanced targeted agents.

## ACKNOWLEDGMENTS

This work was supported by grants from the National Natural Science Foundation of China (Nos. 82170181, 82370188), and Beijing Physician Scientist Training Project (BJPSTP-2024-01) to Liang Wang.
